# Secular increase in the prevalence of gestational diabetes and its associated adverse pregnancy outcomes from 2014 to 2021 in Hebei province, China

**DOI:** 10.3389/fendo.2022.1039051

**Published:** 2022-11-03

**Authors:** Mei-Ling Tian, Li-Yan Du, Guo-Juan Ma, Ting Zhang, Xu-Yuan Ma, Ying-Kui Zhang, Zeng-Jun Tang

**Affiliations:** ^1^ Department of Obstetrics and Gynecology, Hebei General Hospital, Shijiazhuang, China; ^2^ Department of Information Management, Hebei Center for Women and Children’s Health, Shijiazhuang, China; ^3^ Department of Reproductive Medicine, Hebei Reproductive Health Hospital, Shijiazhuang, China

**Keywords:** gestational diabetes mellitus, adverse pregnancy outcomes, Hebei, maternal, offspring

## Abstract

**Objective:**

We aimed to investigate the secular prevalence of gestational diabetes mellitus (GDM) and evaluate its adverse pregnancy outcomes among pregnant women in Hebei province, China.

**Methods:**

We analyzed the data from the monitoring information management system for pregnant women in 22 hospitals of Hebei province, China. In this study, 366,212 individuals with singleton live births from 2014 to 2021 were included, of whom 25,995 were diagnosed with gestational diabetes. We described the incidence of common complications and further analyzed the clinical characteristics in GDM patients and the relationship between GDM and adverse pregnancy outcomes.

**Results:**

The top 3 pregnancy complications in Hebei province are anemia, gestational hypertension, and GDM. The average incidence of GDM was 7.10% (25,995/366,212). The incidence rate of GDM significantly increased from 2014 to 2021 (χ^2^_trend_ = 7,140.663, P < 0.001). The top 3 regions with GDM incidence were Baoding (16.60%), Shijiazhuang (8.00%), and Tangshan (3.80%). The incidence of GDM in urban pregnant women (10.6%) is higher than that in rural areas (3.7%).The difference between the GDM and Non-GDM groups was statistically significant in terms of maternal age, gravidity, parity, education level, and incidence of pregnancy complications (gestational hypertension, heart diseases, and anemia) (P < 0.05). GDM individuals were at significantly increased risk of most assessed adverse pregnancy outcomes, including premature delivery, Cesarean delivery, uterine inertia, neonatal intensive care unit (NICU) admission, Apgar (activity-pulse-grimace-appearance-respiration) score at 1 min, and macrosomia (P < 0.05). The multivariate logistic regression analysis showed that GDM was an independent risk factor in terms of premature birth, Cesarean delivery, uterine inertia, placental abruption, NICU admission, and macrosomia.

**Conclusion:**

The risk of adverse pregnancy outcome in pregnant women with GDM is significantly increased. In order to reduce the occurrence of adverse pregnancy outcomes, effective interventions are needed.

## Introduction

Gestational diabetes mellitus (GDM) is one of the most common complications during pregnancy and is defined as carbohydrate intolerance of any degree with onset or first recognition during pregnancy (1).The prevalence of GDM varies substantially worldwide, ranging from 1% to >30% and continued to increase during the past few decades (2). The incidence of GDM in China was reported to be 11.91% (3). Women with GDM are more likely to experience an adverse outcome, including Cesarean delivery, preeclampsia, soft issue injury of the birth canal, and severe maternal morbidity (4). An increasing number of studies have shown that women with a history of GDM are at an increased risk of being diagnosed as having type 2 diabetes (5). Moreover, long-term complications of mothers with GDM also include cardiovascular disease and other metabolic diseases (6). Furthermore, the incidence of neonatal abortion, stillbirth, macrosomia, neonatal respiratory distress syndrome, neonatal intensive care unit (NICU) admission, and hypoglycemia of newborns in pregnant women with gestational diabetes increased (7). In addition to the short-term perinatal consequences associated with GDM, there are long-term complications for newborns. Studies have found that the risks of obesity, metabolic syndrome, type 2 diabetes, and impaired insulin sensitivity and secretion in offspring of mothers with GDM are 2- to 8-fold of those in offspring of mothers without GDM (4).

The GDM diagnosis is associated with both immediate and long-term adverse consequences for both mother and her offspring. Therefore, we set out to investigate the secular prevalence of GDM and better assess its adverse pregnancy outcomes with gestational diabetes changes from 2014 to 2021 in Hebei province, China.

## Materials and methods

### Study area

Hebei province is located between 113° 27’ and 119°50’E and 36°05’ and 42°40’N and located in the Bohai Sea to the east, Taihang Mountains to the west, and Yanshan Mountains to the north. It covers an area of 188,800 km^2^. It has jurisdiction over 11 prefecture-level cities including Shijiazhuang, Tangshan, and Handan. Hebei province is the only province in China with plateau, mountain, hill, plain, lake, and seashore. The region is an important grain- and cotton-producing area in China. In 2021, the total permanent population of the province will be 74.48 million, and the gross domestic product (GDP) will reach 4,039.13 billion yuan.

### Data collection

**Figure 1:**
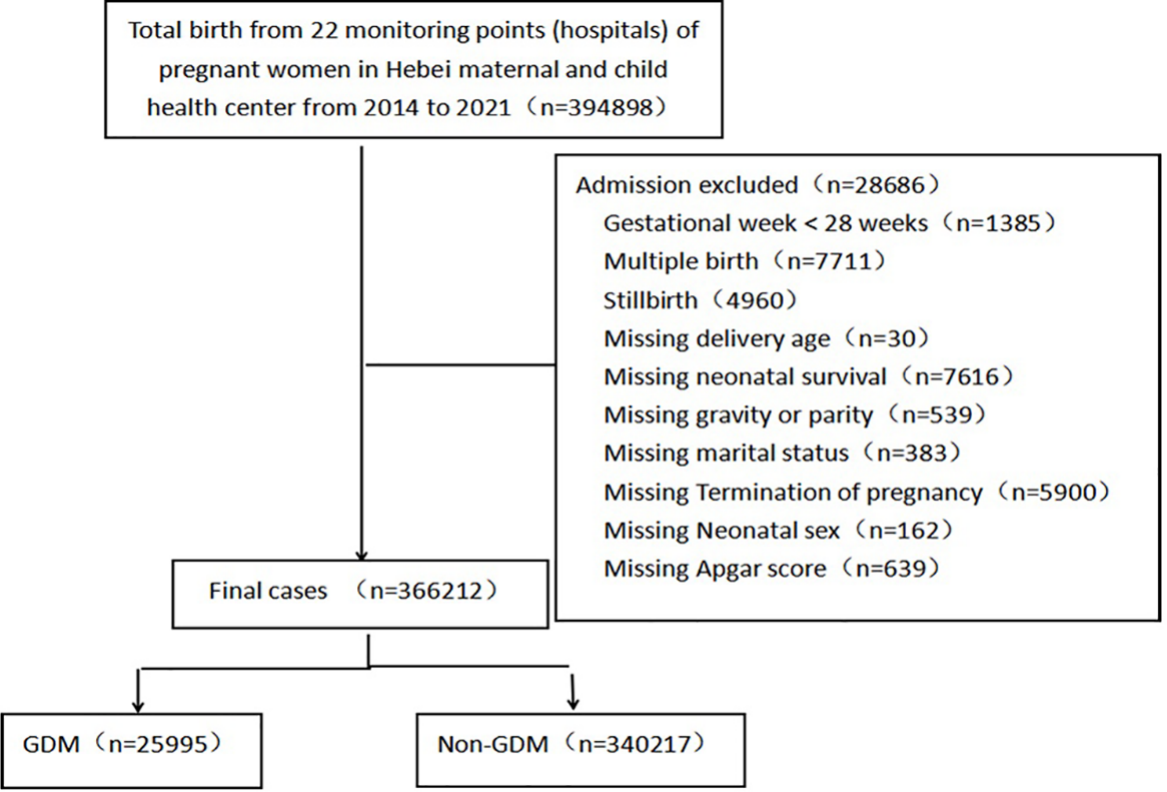
The flow chart of cases enrollment.

This study retrospectively collected 394,898 delivery data from 1 January 2014 to 31 December 2021 from the monitoring information management system for pregnant women in 22 hospitals of Hebei province, China. We acquired informed consent from all subjects. The inclusion criteria covered delivery over 28 weeks and singleton live birth. Exclusion criteria included stillbirth, multiple births, pre-gestational diabetes, and incomplete data. A total of 366,212 deliveries were included, of which 25,995 were diagnosed with GDM. The flowchart of case registration was shown in **Figure 1**.

### Diagnostic approaches and criteria

Pregnant women at 24–28 weeks of gestation were tested for fasting 75-g oral glucose tolerance. GDM was diagnosed if one or more thresholds are met or exceeded: fasting blood glucose 92 mg/dl (5.1 mmol/L), 1-h blood glucose 180 mg/dl (10.0 mmol/L), and 2-h blood glucose 153 mg/dl (8.5 mmol/L) (8).

Macrosomia: a birth weight greater than 4,000 g.

Preterm birth: any birth at less than 37 weeks based on the best obstetric estimate.

### Statistical analyses

The continuous data were tested for normality using the Kolmogorov–Smirnov test. The data description was presented as mean ± standard deviation (mean ± SD) or median [interquartile range (IQR)] for continuous variables and percentages for categorical variables. The counting data are expressed in percentage (%). T-test is used for the comparison between groups of measurement data that conform to normal distribution, F-test is used for the comparison between groups of counting data that do not conform to normal distribution, andχ^2^ test is used for the comparison between groups of counting data. Taking the occurrence of adverse maternal and infant outcomes as the dependent variable, the multivariate logistic regression model was used to analyze the risk factors after adjusting for the confounding factors. All statistical tests of hypotheses will be two-sided, and the criterion for statistical significance is α = 0.05. Statistical analyses were done with SPSS version 17.0 software (IBM).

## Results

### Maternal characteristics

**Figure 2:**
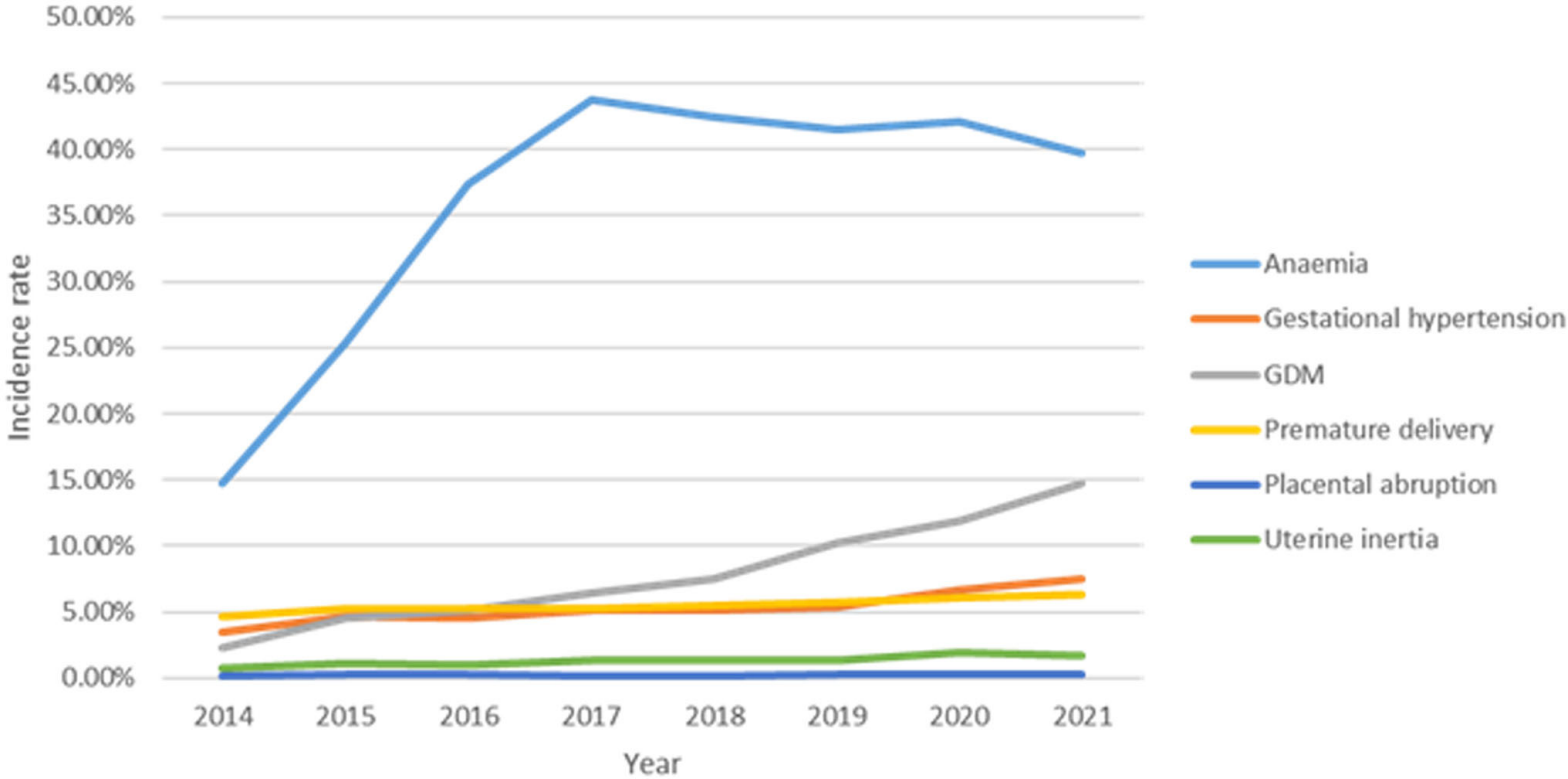
The prevalence of pregnant individuals with pregnancy complications from 2014 to 2021.

After excluding the deliveries that met the exclusion criteria, there were a total of 366,212 deliveries left from the monitoring information management system for pregnant women in 22 hospitals of Hebei province, China, from 2014 to 2021. The top 3 pregnancy complications in Hebei province are anemia, gestational hypertension, and GDM, all of which had an increasing trend. In addition, 25,995 individuals were diagnosed as having GDM. The average incidence of GDM was 7.10% (25,995/366,212). The incidence rate of GDM significantly increased from 2014 to 2021 (χ^2^ trend = 7,140.663, P < 0.001) (**Figure 2**). In the study population, the median age of the GDM group was 30 years (IQR, 27–33 years). The top 3 regions with GDM incidence were Baoding (16.60%), Shijiazhuang (8.00%), and Tangshan (3.80%). The incidence of GDM in urban pregnant women (10.6%) is higher than that in rural areas (3.7%). The demographic and obstetric difference between the two groups was statistically significant in terms of hospital grade, region, maternal age, gravidity, parity, education level, times of prenatal examination, and the incidence of pregnancy complications (heart diseases and anemia) (P < 0.05). There was no significant difference in marital status (P > 0.05) (**Table 1**).

**Table 1:** Maternal characteristics of individuals with singleton live births and GDM in Hebei from 2014 to 2021.

Characteristics	GDM	Control group (n=340,217)	F/^2^	P
	(n=25,995)		
Age	30.0 (27.0, 33.0)	28.0 (26.0, 31.0)	3,510.30	<0.001
Area			16,919.79	<0.001
Baoding	11,722 (16.6%)	58,894 (83.4%)		
Chengde	278 (2.8%)	9,510 (97.2%)		
Shijiazhuang	12,143 (8.0%)	139,918 (92.0%)		
Tangshan	627 (3.8%)	15,996 (96.2%)		
Xingtai	152 (0.7%)	20,674 (99.3%)		
Qinhuangdao	371 (1.5%)	24,322 (98.5%)		
Zhangjiakou	109 (0.7%)	16,159 (99.3%)		
Cangzhou	72 (0.7%)	9,846 (99.3%)		
Hengshui	160 (1.0%)	15,233 (99.0%)		
Handan	361 (1.2%)	29,665 (98.8%)		
**Region**				
Urban	19,200 (10.6%)	161,169 (89.4%)	6,779.02	<0.001
Rural	6,795 (3.7%)	179,048 (96.3%)		
**Hospital levels**			6,872.80	<0.001
Primary	34 (0.7%)	4,697 (99.3%)		
Secondary	6,733 (3.7%)	174,190 (96.3%)		
Tertiary	19,228 (10.6%)	161,330 (89.4%)		
**Age (years)**			1,714.49	<0.001
<18	23 (2.9%)	760 (97.1%)		
18-35	20,951 (6.5%)	302,426 (93.5%)		
35-40	4,239 (11.8%)	31,774 (88.2%)		
≥40	782 (12.9%)	5,257 (87.1%)		
**Marital Status**			2.355	0.125
Single	116 (8.1%)	1,309 (91.9%)		
Married	25,879 (7.1%)	338,908 (92.9%)		
**Education**			1,532.87	<0.001
University and above	13,187 (9.2%)	130,195 (90.8%)		
Junior and Senior Secondary	12,427 (5.8%)	203,137 (94.2%)		
Primary and illiterate	381 (5.2%)	6,885 (94.8%)		
**Gravidity (times)**				
1	7,853 (6.0%)	122,484 (94.0%)	353.425	<0.001
≥2	18,142 (7.7%)	217,733 (92.3%)		
**Parity (times)**				
Primipara	14,069 (5.9%)	225,304 (94.1%)	1,562.22	<0.001
Multipara	11,926 (9.4%)	114,913 (90.6%)		
**Prenatal examination (times)**				
≤3	767 (3.3%)	22,619 (96.7%)	2,352.64	<0.001
04-Jul	9,768 (5.5%)	166,301 (94.5%)		
≥8	15,460 (9.3%)	151,297 (90.7%)		
**Complications during pregnancy**				
**Heart diseases**			23.356	<0.001
Yes	95 (11.4%)	739 (88.6%)		
No	25,900 (7.1%)	339,478 (92.9%)		
**Anemia**			107.086	<0.001
Yes	9,917 (7.7%)	118,972 (92.3%)		
No	16,078 (6.8%)	221,245 (93.2%)

GDM, gestational diabetes mellitus.

### Risk of adverse outcome by gestational diabetes mellitus

GDM individuals were at significantly increased risk of most assessed adverse pregnancy outcomes, including premature delivery, Cesarean delivery, uterine inertia, and gestational hypertension (P < 0.05). There was no statistical difference in puerperal infection, infection of Cesarean section, and placental abruption (P > 0.05). For infant outcomes, there are significant differences in the term of NICU admission, Apgar score at 1 min, and macrosomia (P < 0.05). There was no significant difference in sex of newborn, Apgar score at 5 min, and Apgar score at 10 min between the two groups (**Table 2**).

**Table 2:** Adverse outcome of individuals with GDM in Hebei from 2014 to 2021.

	GDM	Control group	F/^2^	P
	(n=25,995)	(n=340,217)	
**Maternal**				
Premature delivery	2,112 (8.1%)	17,792 (5.2%)	393.807	<0.001
Delivery mode			631.472	<0.001
Spontaneous delivery	15,470 (59.5%)	174,983 (51.4%)		
Cesarean section	10,525 (40.5%)	165,234 (48.6%)		
Uterine inertia	608 (2.3%)	3,949 (1.2%)	272.791	<0.001
Puerperal infection	8 (0.0%)	66 (0.0%)	1.547	0.214
Infection of Cesarean section	2 (0.0%)	20 (0.0%)	0.132	0.716
Placental abruption	79 (0.3%)	844 (0.2%)	2.994	0.084
Gestational hypertension	2,803 (10.8%)	15,758 (4.6%)	1,899.06	<0.001
**Neonate**				
**Gender**			0.408	0.523
Male	13,328 (51.3%)	173,735 (51.1%)		
Female	12,667 (48.7%)	166,482 (48.9%)		
**Apgar score**				
1 min	10.0 (10.0, 10.0)	10.0 (10.0, 10.0)	153.922	<0.001
5 min	10.0 (10.0, 10.0)	10.0 (10.0, 10.0)	1.161	0.281
10 min	10.0 (10.0, 10.0)	10.0 (10.0, 10.0)	0.437	0.508
Admission to NICU	116 (0.4%)	1,665 (0.5%)	0.929	0.335
Macrosomia	2,389 (9.2%)	22,608 (6.6%)	245.956	<0.001

GDM, gestational diabetes mellitus; NICU, neonatal intensive care unit.

**Figure 3:**
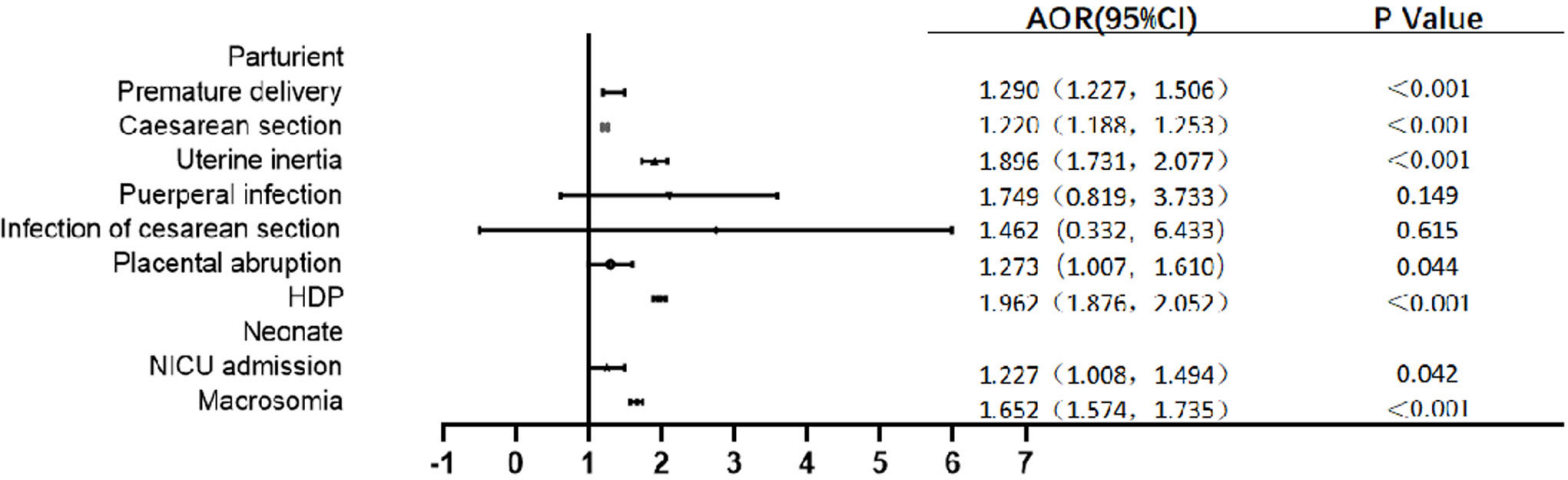
Multivariate logistic regression analysis model of maternal and infant outcomes of GDM individuals. HDP: hypertension during pregnancy.Adjusted confounding factors include age, gravidity, parity, education level, gestation-induced hypertension and anemia.

Univariate logistic regression analysis showed that GDM was a risk factor of premature birth, Cesarean delivery, uterine inertia, gestational hypertension, and macrosomia (P < 0.05), but GDM was not associated with the risk of puerperal infection, infection of Cesarean section, and placental abruption (P > 0.05) (**Table 3**). After adjusting for age, gravidity, parity, education level, and gestation-induced hypertension and anemia, the multivariate logistic regression analysis model showed that the incidence of premature birth in GDM women was 1.290 times higher than that in non-GDM women [odds ratio (OR) = 1.290, 95% CI: 1.227~1.506]. GDM individuals were also at significantly increased risk of most adverse pregnancy outcomes, including Cesarean delivery (OR = 1.222, 95% CI: 1.188~1.253), uterine inertia (OR = 1.896, 95% CI: 1.731~2.077), placental abruption (OR = 1.273, 95% CI: 1.007~1.610), gestational hypertension (OR = 1.962, 95% CI: 1.876~2.052), NICU admission (OR = 1.227, 95% CI: 1.008~1.494), and macrosomia (OR = 1.652, 95% CI: 1.574~1.735) compared with non-GDM individuals. The analyses showed no difference in puerperal infection and infection of Cesarean section between the two groups (**Figure 3**).

**Table 3:** Univariate logistic regression analysis model of maternal and infant outcomes of GDM individuals.

Factors	OR (95% CI)	P值
**Maternal**		
Premature delivery	1.603 (1.529, 1.680)	<0.001
Cesarean section	1.338 (1.353, 1.424)	<0.001
Uterine inertia	2.039 (1.871, 2.223)	<0.001
Puerperal infection	1.587 (0.762, 3.305)	0.214
Infection of Cesarean section	1.309 (0.306, 5.600)	0.716
Placental abruption	1.226 (0.973, 1.544)	0.084
Gestational hypertension	2.489 (2.385, 2.596)	<0.001
**Neonate**		
Admission to NICU	0.911 (0.755, 1.101)	0.335
Macrosomia	1.422 (1.360, 1.486)	<0.001

GDM, gestational diabetes mellitus; NICU, neonatal intensive care unit; OR, odds ratio.

## Discussion

As a special physiological period for women, pregnancy has undergone great changes in its physiology, including reproductive system, endocrine system, and vascular system. As a touchstone during pregnancy, it may trigger potential abnormal glucose and lipid metabolism and then cause metabolic diseases during pregnancy. The top 3 pregnancy complications in Hebei province are anemia, gestational hypertension, and GDM. As a common disease of glucose metabolism in pregnancy, GDM is induced by insulin resistance and pancreatic β-cell dysfunction during pregnancy, which is a transitory form of diabetes. The documented prevalence of GDM varies substantially worldwide, ranging from 1% to >30%. In this study, the average incidence of GDM was 7.10%. The incidence of GDM increased from 2014 to 2021 in Hebei province. Studies have identified a number of GDM risk factors, such as advanced maternal age, previous history of GDM, ethnicity, multiparity, multiple births, genetic heritability, and family history of type 2 diabetes mellitus (2, 3, 9, 10). In this study, we demonstrate that rural, advanced maternal age, high educational level, lack of prenatal examination time, and multiparity are associated with the occurrence of GDM. Therefore, we should do early prevention, detection, and intervention in the management of blood glucose in the advanced maternal age and multiparous individuals, improve the level of grassroots doctors in the screening and management of GDM, and strengthen the publicity of the importance of perinatal health care for township personnel to avoid the occurrence of GDM.

GDM has been identified as one of the major obstacles in achieving improved maternal and child health (11–13). For maternal adverse outcomes in this study, we found that GDM was an independent risk factor for premature birth, Cesarean delivery, uterine inertia, placental abruption, and gestational hypertension. Later retrospective and prospective observational studies indicated that GDM was indeed associated with poor maternal outcomes (preeclampsia, polyhydramnios, operative delivery, shoulder dystocia, birth canal lacerations, etc.) (2, 4, 14). Therefore, we should identify high-risk factors of premature delivery and placental abruption in GDM pregnant women early to avoid the occurrence of adverse outcomes. Moreover, standardizing the diet and weight management of GDM pregnant women can reduce Cesarean section caused by macrosomia. During delivery, we should pay attention to the diet of GDM pregnant women to avoid uterine weakness caused by an insufficient diet. In addition, we should also be alert to placental abruption during delivery. Studies suggested that hyperglycemia is known to increase the risk of preeclampsia (7, 15). Abnormal lipid metabolism in patients with hypertension can easily be complicated by abnormal glucose metabolism. Previous studies have shown that gestational diabetes may be related to puerperal infection and Cesarean section incision infection (16–18). However, this study showed that the incidence of puerperal infection and Cesarean section infection in pregnant women with GDM was not different from that in the non-GDM group. The possible reasons are as follows: The management of GDM patients in the perinatal nutrition outpatient department makes the blood sugar of patients stable in the perinatal period and the use of antibiotics and enhancement of postpartum nursing consciousness reduced the incidence of infection.

In addition to being associated with adverse maternal outcomes, GDM is associated with adverse fetal outcomes. In this study, we found that GDM was an independent risk factor for NICU admission and macrosomia. This is consistent with previous literature reports (12, 19). The rise of maternal blood glucose will cause hyperinsulinemia in the fetus, and insulin cannot be transported through the placenta, which will promote the deposition of liver glycogen, protein synthesis, and fat deposition, thus promoting the growth and development of the fetus. However, the development of each organ of the fetus is asymmetric, and the volume of insulin-sensitive tissues, such as fat and muscle, increases, but the volume of the brain and kidney does not increase, resulting in poor tolerance of the fetus to hypoxia *in utero* and poor adaptability to the external environment, resulting in increased complications of newborns (20). Maternal GDM can also lead to fetal hypoglycemia in the immediate postpartum period when the newborn is still hyperinsulinemic, and this may in turn lead to an increase in NICU admission (21). Moreover, GDM can increase the risk of respiratory distress in the newborn infant that again can lead to an increase in NICU admission (22). The instability of blood glucose is related to the increase in NICU occupancy rate of macrosomia and neonates (20). Health education, medical nutrition treatment, exercise guidance, and blood glucose monitoring are important interventions to stabilize the blood glucose of GDM pregnant women. However, the standardized questionnaire did not include the survey of intervention measures, so we cannot make an analysis on this part of the content. However, with the promotion of our guide and the GDM training program, our clinicians’ diagnosis and treatment level is constantly improving. In the early stage, we will guide and intervene in patients with GDM risk factors to avoid the occurrence of GDM. For pregnant women diagnosed with GDM, we carry out nutrition education and sports guidance and take measures to manage the blood sugar of GDM pregnant women during labor to ensure that the blood sugar of GDM pregnant women is stable, so as to reduce the occurrence of adverse pregnancy outcomes. Moreover, a study had found that fetal sex may influence maternal glucose metabolism in pregnancy. A male fetus is associated with poorer β-cell function, higher postprandial glycemia, and an increased risk of GDM in the mother (23). However, in our study, there was no statistical difference in fetal sex between two groups. The differences in research results may be related to race and the diagnostic criteria of GDM.

## Conclusions

GDM constitutes a significant global health burden, which remains to be the most common metabolic disturbance during pregnancy. Increasing evidence suggests that GDM has significant short- and long-term complications for both the mother and the offspring. There is a high need to prioritize preventive healthcare for pregnant women at risk for GDM. In addition, for GDM individuals, we should achieve standardized management to achieve the goal of stable blood sugar, so as to reduce maternal and infant adverse outcomes. This study only evaluates recent complications, and we will further track long-term effects. This study has a large amount of data and high reliability of the experimental results.

## Data availability statement

The original contributions presented in the study are included in the article/supplementary material. Further inquiries can be directed to the corresponding author.

## Ethics statement

All participants consented in writing to participation, and the above protocols were approved by the ethics committee of Hebei Women and Children’s Health Center.

## Author contributions

M-LT and L-YD designed this study, analyzed the data, and wrote the manuscript. G-JM, TZ, and X-YM contributed to the analysis of the data, Y-KZ and Z-JT reviewed the manuscript. All authors contributed to the article and approved the submitted version.

## Funding

This work was supported by the Youth Science and technology project of Hebei Health Commission (grant number: 20190294、20200001).

## Acknowledgments

We thank the patients, their families, and the investigators who participated in this trial.

## Conflict of interest

The authors declare that the research was conducted in the absence of any commercial or financial relationships that could be construed as a potential conflict of interest.

## Publisher’s note

All claims expressed in this article are solely those of the authors and do not necessarily represent those of their affiliated organizations, or those of the publisher, the editors and the reviewers. Any product that may be evaluated in this article, or claim that may be made by its manufacturer, is not guaranteed or endorsed by the publisher.

## References

[1] Diagnostic criteria and classification of hyperglycaemia first detected in pregnancy: a world health organization guideline. diabetes research and clinical practice. Diabetes Res Clin Pract (2014) 103(3):341–63. doi: 10.1016/j.diabres.2013.10.012 24847517

[2] McIntyre HD Catalano P Zhang C Desoye G Mathiesen ER Damm P. Gestational diabetes mellitus. Nat Rev Dis Primers (2019) 5(1):47. doi: 10.1038/s41572-019-0098-8 31296866

[3] Juan J Yang H. Prevalence, prevention, and lifestyle intervention of gestational diabetes mellitus in China. Int J Environ Res Public Health (2020) 17(24):9517. doi: 10.3390/ijerph17249517 PMC7766930 33353136

[4] Moon JH Jang HC. Gestational diabetes mellitus: Diagnostic approaches and maternal-offspring complications. Diabetes Metab J (2022) 46(1):3–14. doi: 10.4093/dmj.2021.0335 35135076 PMC8831816

[5] Moon JH Kwak SH Jang HC. Prevention of type 2 diabetes mellitus in women with previous gestational diabetes mellitus. Korean J Intern Med (2017) 32(1):26–41. doi: 10.3904/kjim.2016.203 28049284 PMC5214732

[6] Lopez-Tinoco C Roca M Fernandez-Deudero A Garcia-Valero A Bugatto F Aguilar-Diosdado M. Cytokine profile, metabolic syndrome and cardiovascular disease risk in women with late-onset gestational diabetes mellitus. Cytokine (2012) 58(1):14–9. doi: 10.1016/j.cyto.2011.12.004 22200508

[7] Lin J Jin H Chen L. Associations between insulin resistance and adverse pregnancy outcomes in women with gestational diabetes mellitus: a retrospective study. BMC Pregnancy Childbirth (2021) 21(1):526. doi: 10.1186/s12884-021-04006-x 34301212 PMC8306365

[8] Garrison A. Screening, diagnosis, and management of gestational diabetes mellitus. Am Family Physician (2015) 91(7):460–7. 25884746

[9] Kahveci B Ekinci YD. Evaluation of the relationship between HbA1c level and retina choroidal thickness in patients with gestational diabetes mellitus. Arquivos brasileiros oftalmologia (2021) 85(4):339–43. doi: 10.5935/0004-2749.20220045 PMC11878395 34586246

[10] Kahveci B Melekoglu R Evruke IC Cetin C. The effect of advanced maternal age on perinatal outcomes in nulliparous singleton pregnancies. BMC Pregnancy Childbirth (2018) 18(1):343. doi: 10.1186/s12884-018-1984-x 30134873 PMC6106883

[11] Alejandro EU Mamerto TP Chung G Villavieja A Gaus NL Morgan E. Gestational diabetes mellitus: A harbinger of the vicious cycle of diabetes. Int J Mol Sci (2020) 21(14):5003. doi: 10.3390/ijms21145003 PMC7404253 32679915

[12] Murray SR Reynolds RM. Short- and long-term outcomes of gestational diabetes and its treatment on fetal development. Prenat Diagn (2020) 40(9):1085–91. doi: 10.1002/pd.5768 32946125

[13] Vounzoulaki E Khunti K Abner SC Tan BK Davies MJ Gillies CL. Progression to type 2 diabetes in women with a known history of gestational diabetes: systematic review and meta-analysis. BMJ (2020) 369:m1361. doi: 10.1136/bmj.m1361 32404325 PMC7218708

[14] Pettitt DJ Knowler WC Baird HR Bennett PH. Gestational diabetes: infant and maternal complications of pregnancy in relation to third-trimester glucose tolerance in the pima indians. Diabetes Care (1980) 3(3):458–64. doi: 10.2337/diacare.3.3.458 7389563

[15] Hedderson MM Ferrara A. High blood pressure before and during early pregnancy is associated with an increased risk of gestational diabetes mellitus. Diabetes Care (2008) 31(12):2362–7. doi: 10.2337/dc08-1193 PMC2584196 18809624

[16] Chen J Wang Z Wu W Chen H Zhong C Liang L. Clinical analysis of 2860 cases of diabetes in pregnancy: a single-center retrospective study. BMC Pregnancy Childbirth (2022) 22(1):418. doi: 10.1186/s12884-022-04712-0 35585514 PMC9118638

[17] Song H Hu K Du X Zhang J Zhao S. Risk factors, changes in serum inflammatory factors, and clinical prevention and control measures for puerperal infection. J Clin Lab Anal (2020) 34(3):e23047. doi: 10.1002/jcla.23047 31883276 PMC7083398

[18] Zhang X Liao Q Wang F Li D. Association of gestational diabetes mellitus and abnormal vaginal flora with adverse pregnancy outcomes. Med (Baltimore) (2018) 97(34):e11891. doi: 10.1097/MD.0000000000011891 PMC6112872 30142788

[19] Retnakaran R Ye C Hanley AJ Connelly PW Sermer M Zinman B. Effect of maternal weight, adipokines, glucose intolerance and lipids on infant birth weight among women without gestational diabetes mellitus. CMAJ: Can Med Assoc J = J l 'Association medicale Can (2012) 184(12):1353–60. doi: 10.1503/cmaj.111154 PMC3447046 22619341

[20] Barrett HL Dekker Nitert M McIntyre HD Callaway LK. Normalizing metabolism in diabetic pregnancy: is it time to target lipids? Diabetes Care (2014) 37(5):1484–93. doi: 10.2337/dc13-1934 24757231

[21] Wendland EM Torloni MR Falavigna M Trujillo J Dode MA Campos MA. Gestational diabetes and pregnancy outcomes–a systematic review of the world health organization (WHO) and the international association of diabetes in pregnancy study groups (IADPSG) diagnostic criteria. BMC pregnancy childbirth (2012) 12:23. doi: 10.1186/1471-2393-12-23 22462760 PMC3352245

[22] Mortier I Blanc J Tosello B Gire C Bretelle F Carcopino X. Is gestational diabetes an independent risk factor of neonatal severe respiratory distress syndrome after 34 weeks of gestation? a prospective study. Arch Gynecol Obstet (2017) 296(6):1071–7. doi: 10.1007/s00404-017-4505-7 28948345

[23] Retnakaran R Kramer CK Ye C Kew S Hanley AJ Connelly PW. Fetal sex and maternal risk of gestational diabetes mellitus: the impact of having a boy. Diabetes Care (2015) 38(5):844–51. doi: 10.2337/dc14-2551 25693837

